# Prokaryotic Expression, Purification, and Biological Properties of a Novel Bioactive Protein (PFAP-1) from *Pinctada fucata*

**DOI:** 10.3390/md22080345

**Published:** 2024-07-27

**Authors:** Peng Liu, Wenyue Li, Jianbing Liu, Xiaojian Mo, Jiaxing Tang, Jiang Lin

**Affiliations:** 1 School of Basic Medicine, Guangxi University of Traditional Chinese Medicine, Nanning 530200, China; 18002265386@163.com (W.L.); ljb2720579770@126.com (J.L.); mxiaojian0901@163.com (X.M.); p1731911050@163.com (J.T.); 2 Guangxi Key Laboratory of Liver and Spleen Visceral Manifestations in Chinese Medicine, Guangxi University of Traditional Chinese Medicine, Nanning 530200, China

**Keywords:** *Pinctada fucata*, bioactive protein, prokaryotic expression, protein purification, antibacterial activity, antioxidant activity, ACE inhibitory peptide, molecular docking

## Abstract

*Pinctada fucata* meat is the main by-product of the pearl harvesting industry. It is rich in nutrition, containing a lot of protein and peptides, and holds significant value for both medicine and food. In this study, a new active protein was discovered and expressed heterogeneously through bioinformatics analysis. It was then identified using Western blot, molecular weight, and mass spectrometry. The antibacterial activity, hemolysis activity, antioxidant activity, and Angiotensin-Converting Enzyme II (ACE2) inhibitory activity were investigated. An unknown functional protein was screened through the Uniprot protein database, and its primary structure did not resemble existing proteins. It was an α-helical cationic polypeptide we named PFAP-1. The codon-optimized full-length *PFAP-1* gene was synthesized and inserted into the prokaryotic expression vector pET-30a. The induced expression conditions were determined with a final isopropyl-β-d-thiogalactoside (IPTG) concentration of 0.2 mM, an induction temperature of 15 °C, and an induction time of 16 h. The recombinant PFAP-1 protein, with low endotoxin and sterility, was successfully prepared. The recombinant PFAP-1 protein exhibited strong antibacterial activity against methicillin-resistant *Staphylococcus aureus* (MRSA) in vitro, and the diameter of the inhibition zone was 15.99 ± 0.02 mm. Its minimum inhibitory concentration (MIC) and minimum bactericidal concentration (MBC) were 37.5 μg/mL and 150 μg/mL, respectively, and its hemolytic activity was low (11.21%) at the bactericidal concentration. The recombinant PFAP-1 protein significantly inhibited the formation of MRSA biofilm and eradicated MRSA biofilm. It also demonstrated potent 1,1-diphenyl-2-picryl-hydrazyl radical (DPPH) scavenging activity with a half-maximal inhibitory concentration (IC_50_) of 40.83 μg/mL. The IC_50_ of ACE2 inhibition was 5.66 μg/mL. Molecular docking results revealed that the optimal docking fraction of PFAP-1 protein and ACE2 protein was −267.78 kcal/mol, with a confidence level of 0.913. The stable binding complex was primarily formed through nine groups of hydrogen bonds, three groups of salt bridges, and numerous hydrophobic interactions. In conclusion, recombinant PFAP-1 can serve as a promising active protein in food, cosmetics, or medicine.

## 1. Introduction

*Pinctada martensii*, also known as *Pinctada fucata*, is the main maricultural shellfish that produces Hepu pearls in southern China [1]. The meat is one of the main by-products after pearling, containing rich protein resources and many bioactive peptides with special physiological activities [2]. According to the literature, a new antioxidant peptide AOP (GAGLPGKRER) composed of 10 amino acids was isolated from the meat of *P. fucata*. It has good free radical scavenging ability and was successfully expressed recombinantly in *Escherichia coli*. The antioxidant activity of the prepared recombinant peptide (DSAOP) was higher than that of its chemically synthesized and naturally sourced counterparts [3,4]. A simulated gastrointestinal digestion method was used to prepare the hydrolyzed polypeptide components of *P. fucata* meat. The S4 polypeptide components (RL, RGL, PR, etc.) exhibited strong antioxidant activity and could protect human hepatocellular carcinoma (HepG2) cells from oxidative damage induced by hydrogen peroxide (H_2_O_2_) [5]. Two active peptides (FRVW and LPYY) found in the meat of *P. fucata* can effectively inhibit ACE and have potential value as a treatment for hypertension [6]. Two new ACE inhibitory peptides (HLHT and GWA) were isolated and purified from the meat protein hydrolysate of *P. fucata*, showing antihypertensive effects on rat hypertension [7]. A new galactose-binding lectin *PFL-96* gene was cloned from the meat of *P. fucata*. The recombinant PFL-96 protein expressed by *E. coli* exhibited significant inhibitory and bactericidal activity against Gram-positive bacteria such as *Bacillus subtilis* and *Staphylococcus aureus*. The inhibitory effect against Gram-negative bacteria was not significant, but it showed a significant inhibitory effect against *Vibrio alginolyticus*. It also demonstrated significant antifungal effects on the fungus *Candida albicans* and had a notable inhibitory effect on the proliferation of Hela, HepG2, and C666-1 tumor cells [8]. In conclusion, the meat protein of *P. fucata* can be utilized as an ideal resource for the development of marine bioactive peptides with antibacterial, antioxidant, and anti-ACE activities.

Compared with land-based peptides, marine peptides have different amino acid sequences, structures, and functions, and are an important source of bioactive peptides [9]. The main strategy for extracting active peptides from marine organisms is to directly grind the tissues, then obtain crude extracts through organic extraction or concentration, screen extracts with specific biological activities, such as antibacterial, antioxidant, anti-tumor, etc., and further separate and purify single active peptides by solid-phase extraction, gel filtration chromatography, and reversed-phase high-performance liquid chromatography. However, this strategy is limited to natural raw materials, and the separation and purification process of active peptides is complicated and expensive. With the continuous development of DNA recombination, genetic engineering technology, and genome sequencing technology, more and more new marine active peptides can be analyzed and mined from genome, transcriptome, and other databases and prepared by recombinant expression technology [10]. For example, hepcidin, an antimicrobial peptide expressed by *E. coli*, has significant antibacterial activity against *Vibrio parahemolyticus*, *E. coli*, and *S. aureus* in vitro [11]. *Terebra guttata*’s venom peptide Tgu6.1, expressed by recombinant *E. coli* deficient in outer membrane protease T (*ompT*), has paralytic activity [12]. The antimicrobial peptides of *Penaeus monodon* expressed in the prokaryotes of *E. coli* showed significant antibacterial activity against tested bacteria [13]. It can be seen that the use of prokaryotic expression of marine bioactive peptides for antibacterial studies is relatively common, but there are few reports on the multifunctional properties of recombinant active peptides. At present, both the genome and proteome databases of *P. fucata* have been published on NCBI [14,15], but many of the proteins are defined as Uncharacterized proteins whose biological functions are unknown, which is equivalent to a treasure house waiting to be excavated. Some conventional bioinformatics tools are helpful to the development and mining of bioactive polypeptides of *P. fucata*. For example, the online prediction tool of the Collection of Anti-Microbial Peptides (CAMP_R3_) facilitates the of antimicrobial peptides [16]. Therefore, in this study, we utilized bioinformatics combined with an RT-PCR strategy for the first time to identify new active proteins in the meat of *P. fucata*. Following heterologous recombinant expression preparation, we aimed to investigate the biological activity of these proteins through antibacterial assays and explore potential antioxidant and antihypertensive effects similar to those of traditional Chinese medicine pearl. DPPH scavenging and hydroxyl free radical scavenging are commonly employed to assess the antioxidant properties of active substances [17]. However, the hydroxyl free radical scavenging kit requires a sample solvent other than phosphoric acid buffer. As the proteins prepared by us were ultimately dialyzed and preserved in phosphate buffer saline (PBS), we chose to evaluate their antioxidant properties based on DPPH scavenging ability. Additionally, ACE inhibitors are considered promising for the treatment of hypertension and related conditions. In recent years, various ACE inhibitors derived from marine invertebrates, such as shrimp, clams, and oysters, have been extensively studied [18]. Therefore, we employed these three screening strategies to identify more active proteins with diverse biological functions from *P. fucata*.

## 2. Results

### 2.1. Bioinformatics and RT-PCR Were Used to Screen the Active Peptides of P. fucata

According to *P. fucata*’s protein database in UniProt (Taxon ID: 50426), we have downloaded a total of 870 protein sequences, which include all the uncharacterized protein sequences within 100 amino acids in length and 3 sequences above 100 amino acids in length. All of these sequences were submitted to CAMP_R3_ for prediction. The results indicated that 62 of them were analyzed by CAMP_R3_ prediction tools (Random Forest classifier, Artificial Neural Network (ANN) classifier, Discriminant Analysis) and were classified as antimicrobial peptides (AMPs). The gene with the highest comprehensive score was selected for reverse transcription-polymerase chain reaction (RT-PCR), and two genes were amplified. The study related to No. 1 gene has been published by us [8], and No. 6 gene is the active peptide in this study (Supplementary Figure S1). The entry number of this protein in the UniProt database is A0A194ALV1. We have renamed the protein PFAP-1.

### 2.2. PFAP-1 Codon Optimization and Gene Synthesis, Prokaryotic Expression Recombinant Plasmid Construction, and Identification

**Figure 1:**
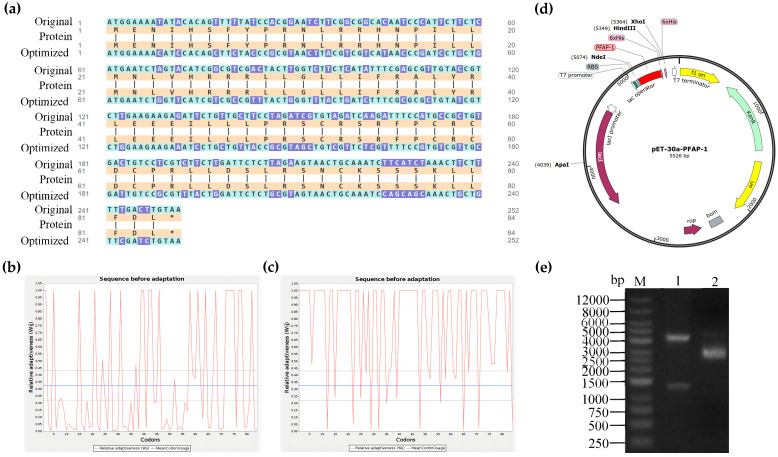
Optimization of the codon of *PFAP-1* gene, construction of recombinant plasmid, and identification. (**a**) *PFAP-1* gene original sequence and codon-optimized sequence, and corresponding amino acid sequence of *P. fucata*. (**b**) The use of the wild-type PFAP-1 gene codon. (**c**) The use of the optimized *PFAP-1* gene codon. (**d**) Schematic diagram of pET-30a-PFAP-1 recombinant plasmid. (**e**) Results of double digestion of the recombinant plasmid. M is DNA marker, and 1 is the product of double enzyme digestion of the recombinant plasmid. 2 is the recombinant plasmid.

By utilizing the Java Codon Adaptation Tool (JCat) analysis, the Codon Adaptation Index (CAI) value of the original *PFAP-1* gene sequence was 0.43, with a GC content of 40.87% and a higher frequency of low-frequency codons. Following optimization with GenSmart™ Codon Optimization (Version Beta 1.0), the CAI improved to 0.85. The GC content increased to 48.81%, which is advantageous for expression in *E. coli* (Figure 1a–c). Subsequently, the gene was chemically synthesized and linked to the pET-30a expression vector to construct the recombinant plasmid PET-30a-PFAP-1 (Figure 1d). Verification of the recombinant plasmid was conducted through double enzyme digestion (*Xho* I and *Apa* I), resulting in two linear fragments of approximately 1325 bp and 4201 bp, respectively (Figure 1e). Sequencing and Blast analysis confirmed the successful construction of the recombinant plasmid pET-30a-PFAP-1.

### 2.3. Bioinformatics Analysis of PFAP-1 Protein

**Figure 2:**
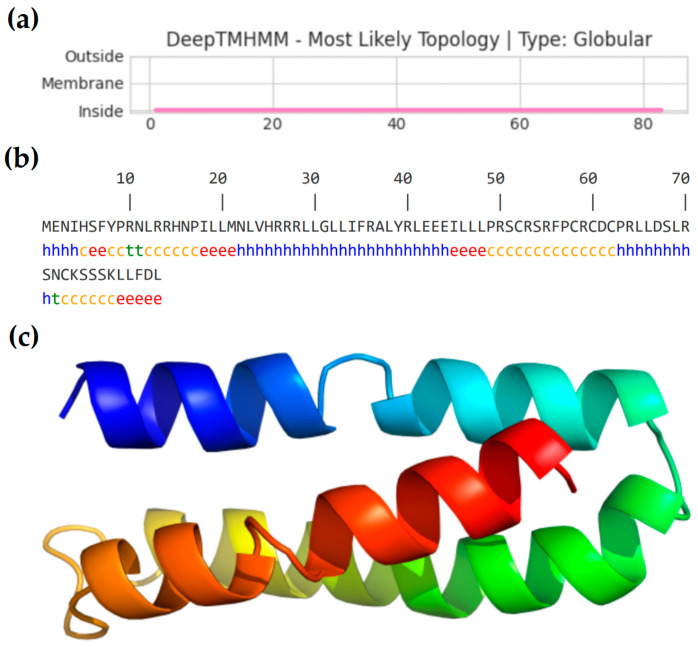
Bioinformatics analysis was conducted on the active protein PFAP-1 of *P. fucata*. (**a**) Prediction of the transmembrane domain of PFAP-1. (**b**) Prediction of the secondary structure of PFAP-1. (**c**) Tertiary structure prediction of PFAP-1.

The PFAP-1 protein consists of 83 amino acid residues, has a molecular weight of about 10.00 kDa, an estimated isoelectric point of 10.40, a total net charge of +9, and a hydrophobicity of 43% (Table 1). DeepTMHMM prediction results showed that the PFAP-1 protein did not contain signal peptides and was an intracellular protein (Figure 2a). SOPMA predicted that the secondary structure of the PFAP-1 protein mainly consisted of α helix (43.37%), random coil (34.94%), extended chain (18.07%), and β turn (3.61%) (Figure 2b). I-TASSER software (version 5.1) was used to predict the tertiary structure of the full length of the PFAP-1 protein, and PyMol software (version 1.7.0.0) was used to show that it was mainly composed of three α-helices located in Glu2-Arg27, Leu29-Ser54, and C62-Asp82 (Figure 2c).

### 2.4. Prokaryotic Expression and Purification of PFAP-1 Protein

**Figure 3:**
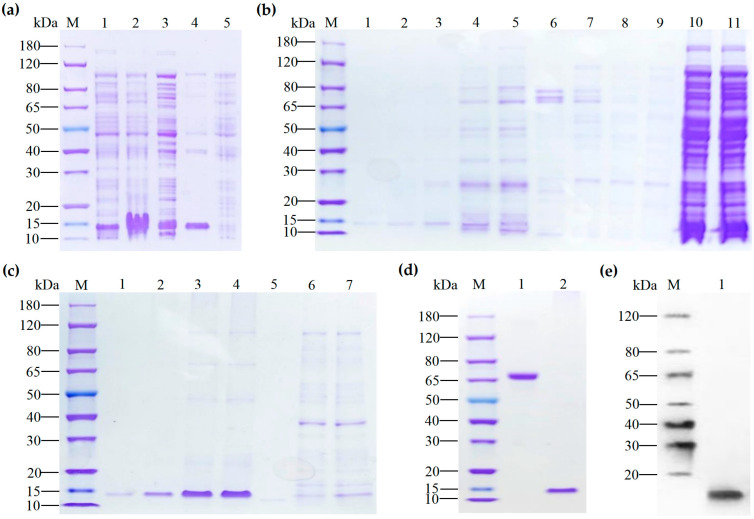
Expression, purification, and Western blot identification of PFAP-1 protein. (**a**) The expression of PFAP-1 protein in BL21(DE3) was analyzed by 12% SDS-PAGE. Lane M: Protein marker; Lane 1: Induction at 15 °C for 16 h; Lane 2: Induced at 37 °C for 16 h; Lane 3: Supernatant after whole bacteria breaking; Lane 4: Precipitate after the whole bacteria breakdown. (**b**) The 12% SDS-PAGE analysis of the supernatant soluble PFAP-1 recombinant protein purification results. Lane M: Protein marker; Lane 1: Supernatant after whole bacteria breaking and centrifugation; Lane 2: Effluent after incubation of supernatant with Ni-IDA; Lanes 3–4: Elution components of Imidazole at 50 mM; Lanes 5–6: Elution components of 100 mM Imidazole; Lanes 7–11: Elution components of Imidazole at 500 mM. (**c**) Purification results of inclusion body PFAP-1 recombinant protein. Lane M: Protein Marker; Lane 1: Supernatant after inclusion body dissolution centrifugation; Lane 2: Effluent after incubation of supernatant with Ni-IDA; Lane 3: Elution component of Imidazole at 50 mM; Lanes 4–7: Elution components of Imidazole at 500 mM. (**d**) Quality inspection results of purified recombinant PFAP-1 protein. Lane 1: BSA (1.00 μg); Lane 2: PFAP-1 protein (0.60 μg). (**e**) WB verification of recombinant PFAP-1 protein. Lane 2: PFAP-1 protein (0.60 μg).

The verified recombinant expression plasmid was transferred into *E. coli* BL21 (DE3), induced by IPTG, and the bacteria were collected for SDS-PAGE analysis. The results showed that PFAP-1 could be significantly expressed under the induction conditions of 15 °C and 37 °C compared with the non-induced group. After the bacteria were crushed by ultrasound, the expression forms were analyzed by SDS-PAGE. The results showed that both the supernatant and precipitation after crushing had the target protein distribution (Figure 3a). However, due to the excessive protein impurities in the supernatant, the target protein was not purified from the supernatant (Figure 3b). Therefore, we decided to purify the target protein from the inclusion body. The result showed that there were obvious bands of recombinant protein expression at the 15 kDa band (Figure 3c). The eluted components with higher purity were collected for renaturation, dialysis, endotoxin removal, and filtration. Finally, the obtained protein concentration was 0.60 mg/mL, and 0.6 µg was used for SDS-PAGE detection. The results showed that there was a specific band at 15 kDa with high purity (Figure 3d). WB verification results showed that there was still a specific band at the same molecular weight position (Figure 3e). The results showed that PFAP-1 recombinant protein with low endotoxin, sterility, and high purity was successfully prepared.

### 2.5. Molecular Weight Determination and Mass Spectrometry Identification of PFAP-1 Recombinant Protein

**Figure 4:**
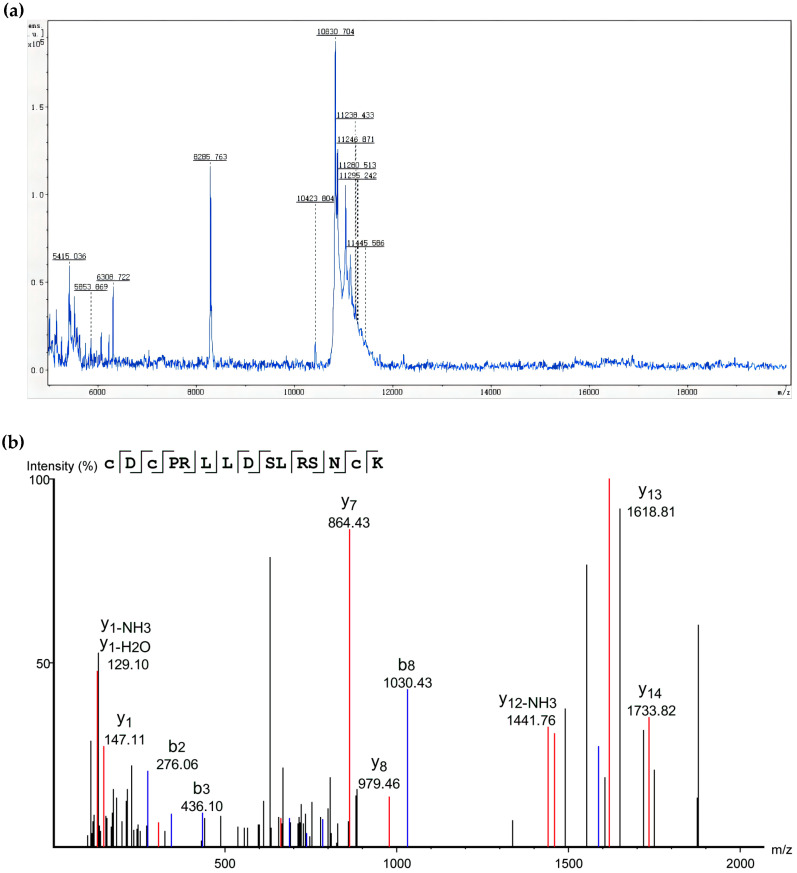
Molecular weight determination and mass spectrometry identification of PFAP-1 recombinant protein. (**a**) Molecular weight determination of PFAP-1 recombinant protein; (**b**) fingerprint of PFAP-1 recombinant protein peptide.

MALDI-TOF-TOF was used to determine the molecular weight of the PFAP-1 recombinant protein. The results indicated a molecular weight of approximately 10,830 Da, which was consistent with the expected value (Figure 4a). The LC-MS method was employed to identify the amino acid sequence of the peptide segment of the PFAP-1 recombinant protease. The results revealed a high degree of matching between the detected fragment ion peak and the theoretical fragment peak. Additionally, the secondary fragment ion peak of ‘CDCPRLLDSLRSNCK’ was displayed in Figure 4b. The purified protein was the PFAP-1 recombinant protein.

### 2.6. Antibacterial Activity of PFAP-1 Recombinant Protein

**Figure 5:**
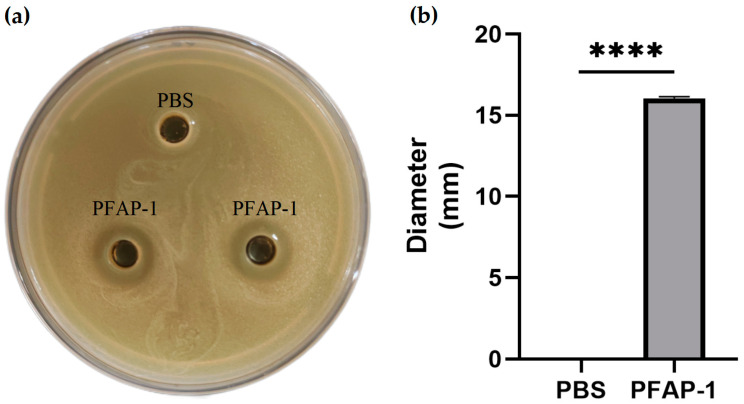
Antibacterial activity of PFAP-1 recombinant protein against MRSA. (**a**) Inhibition zone of PFAP-1 recombinant protein against MRSA; (**b**) statistical results of the antibacterial zone diameter of PFAP-1 against MRSA. **** Represents a significant difference (*p* < 0.0001).

**Figure 6:**
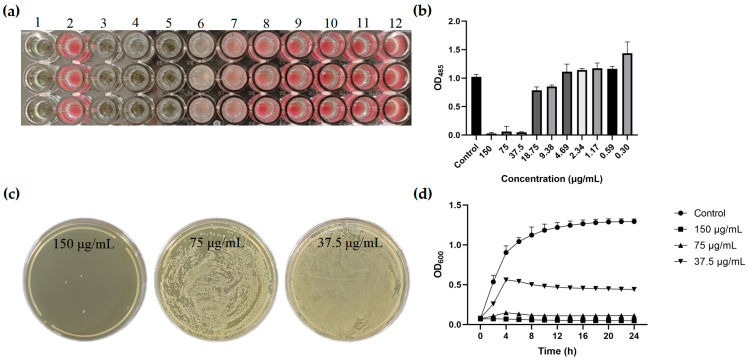
The determination of the MIC and MBC of PFAP-1 recombinant protein on MRSA and its effect on the growth curve of MRSA. (**a**) 2,3,5-Triphenyltetrazolium chloride (TTC) color display diagram, where columns 1–12 indicate that the final concentrations of PFAP-1 recombinant protein in the pore were 0, 150, 75, 37.5, 18.75, 9.38, 4.69, 2.34, 1.17, 0.59, and 0.30 μg/mL; (**b**) OD_485_ absorbance analysis diagram; (**c**) plate colony growth diagram; (**d**) influence of PFAP-1 recombinant protein on the MRSA growth curve.

The Oxford Cup method was used to detect the antibacterial activity of PFAP-1 recombinant protein. The results showed that it had obvious antibacterial activity against MRSA, and its antibacterial zone diameter was 15.99 ± 0.02 mm (Figure 5). The microbroth dilution method was employed to determine the MIC of PFAP-1 recombinant protein on MRSA, revealing a MIC of 37.5 μg/mL (Figure 6a,b). Plate coating results demonstrated that the MBC was 150 μg/mL (Figure 6c). Growth curve analysis revealed that all PFAP-1 recombinant proteins could notably inhibit the growth of MRSA compared to the negative control group. Particularly at concentrations of 1 and 2 × MIC, the growth of MRSA was significantly suppressed (Figure 6d).

### 2.7. Fluorescence Staining Observation

**Figure 7:**
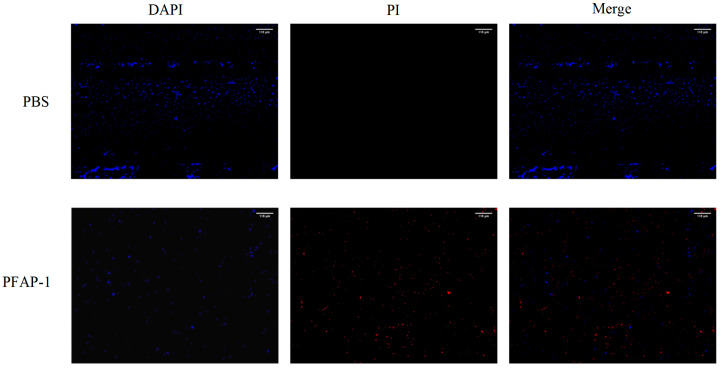
Effect of PFAP-1 recombinant protein on membrane permeability of MRSA. The concentration of PFAP-1 recombinant protein was 1 × MIC (The scale bar is 115 μm).

The impact of PFAP-1 recombinant protein on MRSA cell membrane permeability was examined using fluorescence microscopy. The findings revealed that the control group primarily exhibited blue fluorescence following co-incubation with MRSA, with minimal red fluorescence, suggesting intact MRSA cells. Conversely, the experimental group displayed predominantly red fluorescence and a minor amount of blue fluorescence, indicating that the majority of MRSA cell membranes were compromised (Figure 7).

### 2.8. Scanning Electron Microscopy (SEM) Observation

**Figure 8:**
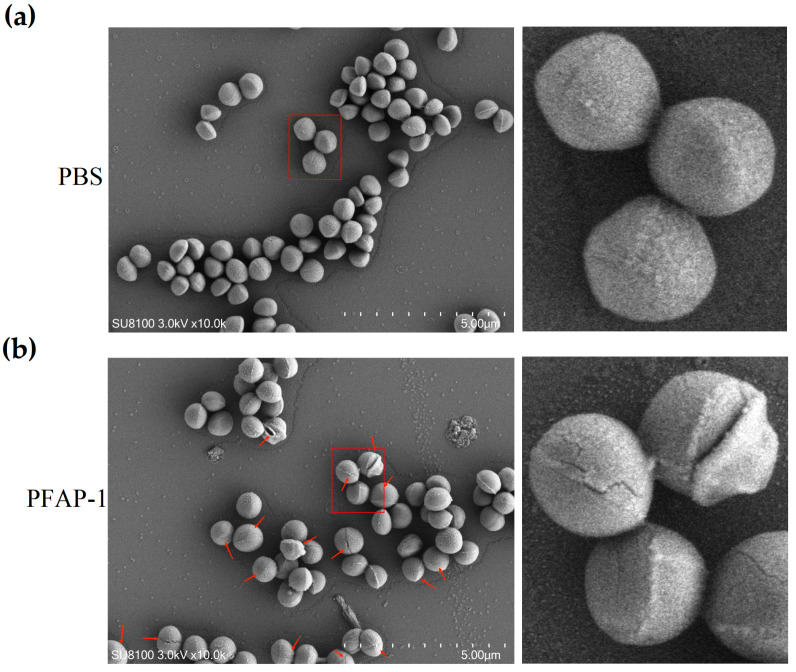
The effect of PFAP-1 on the morphology of MRSA was observed using a scanning electron microscope. (**a**) The impact of PBS on the morphology of MRSA. (**b**) The impact of PFAP-1 on the morphology of MRSA. The red arrows indicate the locations of cracks and grooves.

SEM was utilized to further investigate the impact of PFAP-1 recombinant protein on the microscopic morphology of MRSA. The findings revealed that in the control group, MRSA appeared clear, smooth, and intact, displaying a typical spherical structure (Figure 8a). In the group treated with 1 × MIC of PFAP-1 recombinant protein, MRSA exhibited noticeable cracks and grooves, resulting in a roughened surface (Figure 8b). These results indicate that the PFAP-1 recombinant protein can significantly induce morphological changes in MRSA.

### 2.9. PFAP-1 Recombinant Protein Inhibits Biofilm Activity

**Figure 9:**
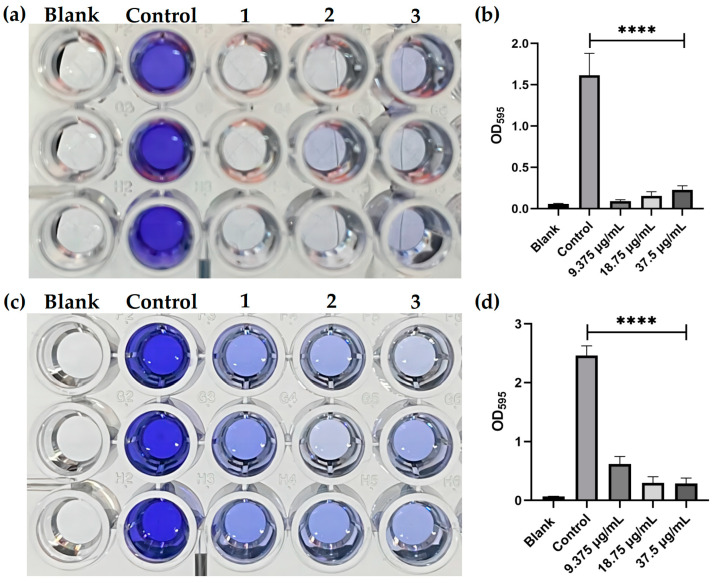
Inhibition of PFAP-1 recombinant protein on the formation of MRSA biofilm and elimination of mature biofilm. (**a**) Inhibition of PFAP-1 recombinant protein on the formation of MRSA biofilm was studied. The control group showed a final concentration of PFAP-1 in the well at 0 μg/mL, while columns 1–3 indicated concentrations of 9.375, 18.75, and 37.5 μg/mL for PFAP-1. (**b**) Statistical results of OD_596_ absorbance. (**c**) The elimination effect of PFAP-1 recombinant protein on MRSA biofilm. (**d**) Statistical results of OD_596_ absorbance. **** Represents a significant difference (*p* < 0.0001).

MRSA in the negative control group could significantly form a biofilm, while the recombinant protein PFAP-1 treatment group could significantly inhibit the formation of MRSA biofilm (Figure 9a,b). Regarding the elimination of mature biofilm, the results indicated that the recombinant protein PFAP-1 had a significant effect on eliminating the mature biofilm of MRSA compared to the negative control group (Figure 9c,d).

### 2.10. Hemolytic Activity of PFAP-1 Recombinant Protein

**Figure 10:**
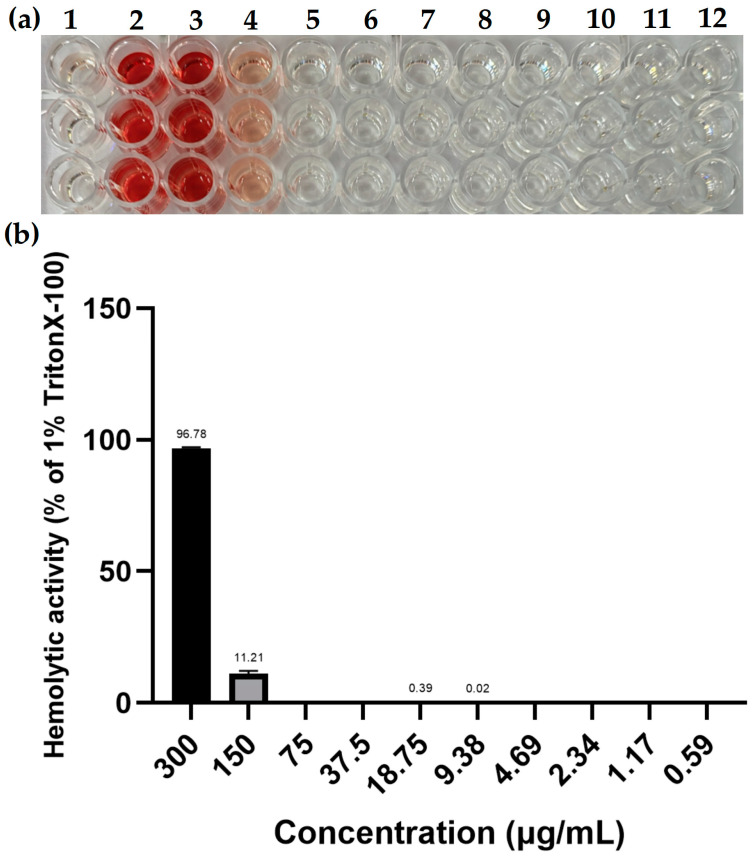
Hemolytic activity of PFAP-1 recombinant protein. (**a**) The hemolysis of 2% rabbit red blood cells under different concentrations of PFAP-1 recombinant protein, where column 1 represents the negative control, column 2 represents the positive control with 1.0% Triton X-100, and the final concentration of PFAP-1 recombinant protein in columns 3–11 was 300, 150, 75, 37.5, 18.75, 9.38, 4.69, 2.34, 1.17, and 0.59 μg/mL, respectively. (**b**) Statistical data on the hemolysis rate of PFAP-1 recombinant protein at various concentrations.

As depicted in the results of the hemolysis experiment (Figure 10), it is evident that at a final concentration of 300 μg/mL of PFAP-1 recombinant protein, the hemolysis rate was high (96.78%). Conversely, at a final concentration of 150 μg/mL, the hemolysis rate was low (11.21%). Moreover, at a final concentration of 75 μg/mL or lower, the hemolytic activity of PFAP-1 recombinant protein was found to be negligible. These findings indicate that PFAP-1 recombinant protein exhibits low hemolytic activity on mammalian blood cells within the effective antibacterial concentration range.

### 2.11. PFAP-1 Recombinant Protein Scavenge DPPH Free Radicals and Inhibit ACE2 Activity

**Figure 11:**
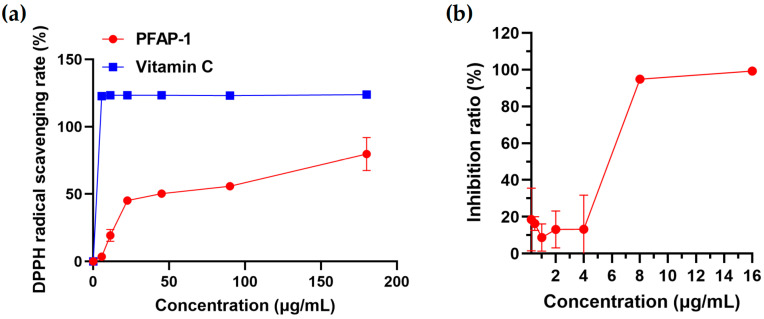
PFAP-1 recombinant protein exhibits scavenging activity against DPPH free radicals and inhibits ACE2 activity. (**a**) The DPPH free radical scavenging activity of PFAP-1 recombinant protein. (**b**) PFAP-1 recombinant protein inhibits ACE2 activity.

Compared with vitamin C, the recombinant protein PFAP-1 exhibits weaker DPPH clearance, with a clearance rate of 79.67% at its highest concentration, and an IC_50_ of 40.83 μg/mL (Figure 11a). Recombinant protein PFAP-1 showed low inhibitory activity against ACE2 in the range of 0.5–4 μg/mL. When its concentration exceeded 4 μg/mL, the inhibitory rate against ACE2 increased significantly, reaching its maximum inhibitory rate at 8–16 μg/mL, with an IC_50_ of 5.66 μg/mL (Figure 11b).

### 2.12. PFAP-1 Protein Docked with ACE2 Molecule

**Figure 12:**
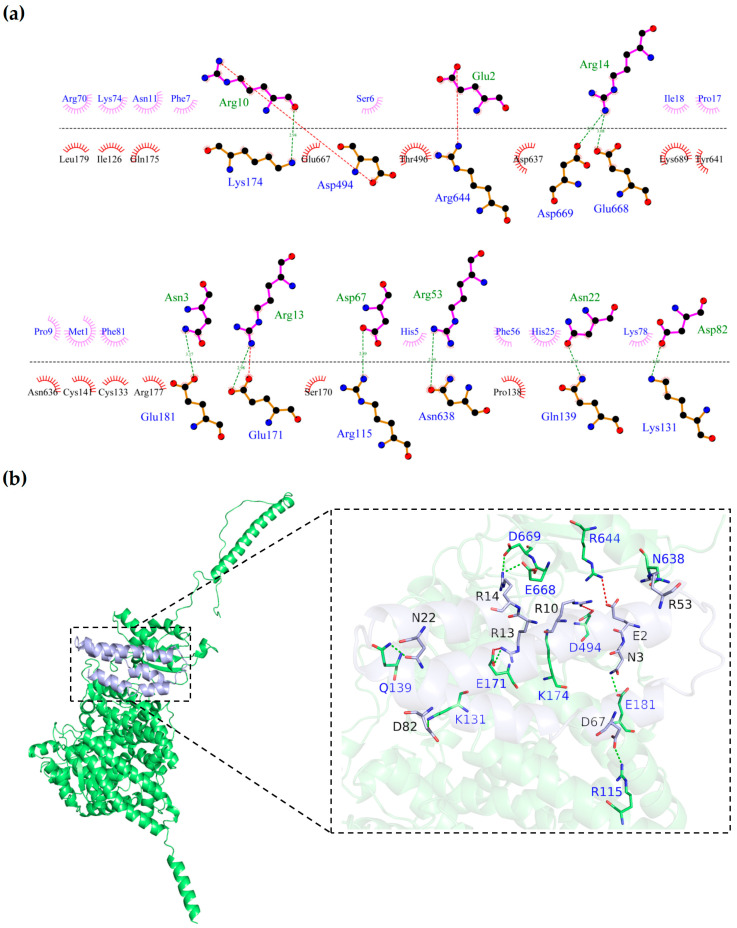
Interaction pattern between PFAP-1 protein and ACE2 protein. (**a**) The 2D interaction pattern between the proteins, where dentate amino acids represent hydrophobic interactions, and the green dotted line indicates hydrogen bonding. (**b**) The 3D interaction patterns between the proteins.

The docking score of PFAP-1 protein and ACE2 protein was −267.78 kcal/mol, and the confidence score was 0.913. The results indicate that the composite model obtained by docking is highly reliable. Subsequently, this study further analyzed the interaction patterns of spatial structure binding sites and binding regions between PFAP-1 protein and ACE2 protein. As shown in Figure 12, nine groups of hydrogen bond interactions and three groups of salt bridge interactions were formed between PFAP-1 protein and ACE2 protein. Among the hydrogen bonds formed by amino acids between the two proteins, the amino acid residues from ACE2 protein include Arg115, Lys131, Gln139, Glu171, Lys174, Glu181, Asp494, Asn638, Arg644, Glu668, and Asp669. Amino acid residues from PFAP-1 protein are Glu2, Asn3, Arg10, Arg13, Arg14, Asn22, Arg53, and Asp67. Additionally, a significant number of hydrophobic interactions between the two proteins promote further stable binding to form a complex.

## 3. Discussion

The ocean is the largest ecosystem on Earth, covering 70% of the Earth’s surface and home to 80% of the world’s organisms [19]. Compared with land, the marine environment is characterized by darkness, high salt, high pressure, low temperature, and hypoxia, which makes marine organisms have different active substances from terrestrial organisms, and have unique structural and functional characteristics [20]. Therefore, marine biological resources are widely exploited in research for drugs, functional foods, cosmetics, and other purposes. Among many marine active substances, marine bioactive peptides show significant diversity in terms of structure and biological activity, and have attracted wide attention in the research and development fields of functional foods, cosmetics, and drugs [10]. The main methods for obtaining marine active peptides include solvent extraction, microbial fermentation, protease hydrolysis, and ultrasonic-assisted extraction, but there are also many challenges and certain limitations in raw materials [21]. In addition, chemical synthesis of short peptides and the preparation of marine bioactive peptides using model biological recombination expression have become important methods for production [22]. Therefore, in this study, a new active protein, PFAP-1, derived from *P. fucata*, was prepared by recombinant expression in *E. coli*, and its antibacterial activity, biofilm inhibitory activity, hemolysis activity, antioxidant activity, and ACE2 inhibitory activity were detected.

There is a significant inequality between gene and protein annotation resources, resulting in many uncharacterized proteins that have not undergone functional studies. However, this does not imply that they are non-functional. For instance, in a human cell line, there are 1878 genes essential for cell proliferation, but 330 of them are associated with proteins that have not been characterized [23,24]. These uncharacterized proteins may potentially harbor crucial active proteins awaiting exploration. Antimicrobial peptides typically consist of fewer than 100 amino acids [25]. Therefore, in the *P. fucata* protein database, the focus was on examining uncharacterized proteins with less than 100 amino acids. The CAMP_R3_ software was utilized to predict whether these proteins might exhibit antimicrobial properties. For example, Yang S. et al. employed CAMP_R3_ and AntiBP online software (version 2) to predict and identify antimicrobial peptide segments from hemoglobin of *Tegillarca granosa*. One of the derived peptides, TGH1 (TWEMVSGKKKNGVVLMIK), demonstrated significant antibacterial activity against *Vibrio parahaemolyticus* [26]. Yang, S. et al. also applied this approach to predict an antimicrobial peptide segment from the hemoglobin of large yellow fish, resulting in the discovery of a new derivative peptide, LCH4 (AWQKFLSAVVSALGR), with notable antibacterial activity against *Staphylococcus aureus* and *Vibrio parahaemolyticus* [27]. Hernandez-Arvizu, E.E. et al. identified a derivative peptide, Vcn-23 (FFKKVKKSVKKRLKKIFKKPMVI), from a cathelicidins-like peptide of *Crotalus aquilus* using CAMP_R3_, which exhibited potent broad-spectrum antibacterial activity [28]. It is evident that bioinformatics tools play a crucial role in the rapid discovery of new antimicrobial peptides. Although 62 potential antimicrobial peptides were predicted by CAMP in this study (Supplementary Table S1), experimental validation is still necessary. Consequently, eight candidate peptides were selected for RT-PCR verification based on the CAMP score ranking. Two genes were identified, one of which was PFAP-1, the focus of this study. Subsequently, further bioinformatics analysis, prokaryotic expression preparation, and antibacterial activity testing were conducted.

Most natural antimicrobial peptides have been reported to have a net charge between +4 and +9 and a hydrophobicity of 40% to 60% [29]. For instance, as reported by Zhang, W. et al., an antimicrobial peptide Scyreptin1-30 from *Scylla paramamosain* has an isoelectric point of 10.05, a hydrophobicity of 40%, and a net charge of +5, and exhibits significant antibacterial activity against *Pseudomonas aeruginosa* [30]. Chen, Y.C. et al. reported that an antimicrobial peptide Sp-LECin from *S. paramamosain* has an isoelectric point of 9.87, a hydrophobicity of 45%, and a net charge of +4, also showing significant antibacterial activity against *P. aeruginata* [31]. In this current study, the number of amino acids of PFAP-1 is 83, the molecular weight is about 10.00 kDa, the isoelectric point is 10.40, the net charge is +9, and the hydrophobicity is 43% (Table 1), which aligns with the basic characteristics of most cationic antimicrobial peptides. Antimicrobial peptides can be categorized into four types based on their structure: α-helix peptides, β-lamellar peptides, linear extension peptides, and peptides with both α-helix and β-lamellar peptides [32]. The secondary and tertiary structure prediction of PFAP-1 indicated that it mainly consisted of α-helix peptides (Figure 2), suggesting that it is an α-helix cationic antimicrobial peptide.

Due to the issue of codon preference, codon optimization is necessary for eukaryotic genes to be effectively expressed in prokaryotes [33]. When the CAI exceeds 0.80, foreign genes can be expressed well in the intended organisms [34]. Following the optimization of the original *PFAP-1* sequence, CAI values increased from 0.43 to 0.85 (>0.8), resulting in a more uniform GC distribution and a significant increase in the ratio of *E. coli* preferred codons. This optimized sequence can be effectively expressed in *E. coli* (Figure 1). The pET series expression plasmids are commonly used in *E. coli* for recombinant protein expression, with pET-28a being the most prevalent. It includes a T7 promoter, adjacent Lac operon sequence, and ribosome binding site (RBS) sequence [35]. Similarly, pET-30a is a highly efficient prokaryotic expression plasmid. Hence, in this study, we utilized pET-30a to construct the recombinant plasmid. The primary reason for this choice is that selecting pET-28a would result in the N-terminal of the recombinant protein containing a lengthy peptide segment encoded by the carrier sequence, leading to the expressed protein’s molecular weight being significantly larger than the theoretical target protein. By using pET-30a, the target protein can be positioned adjacent to the RBS sequence, minimizing the presence of carrier sequence-encoded peptides (Figure 1). The PFAP-1 recombinant protein contains a six-histidine tag, resulting in a slightly larger theoretical molecular weight of 10,832 Da. The matrix-assisted laser desorption ionization-tandem-time-of-flight (MALDI-TOF-TOF) analysis revealed a molecular weight of approximately 10,830 Da, which is in close agreement with the theoretical value. Apart from the main peak at 10,830, additional spectral peaks at 11,238 and 11,445 were observed (Figure 3 and Figure 4). While prokaryotic proteins typically have minimal post-translational modifications, there could be some limited alterations such as phosphorylation, acetylation, etc., which could cause a slight increase in the molecular weight [36,37]. At present, an increasing number of pathogens have developed multi-drug resistance. MRSA is a leading cause of infection globally, resulting in bacteremia, respiratory tract infection, skin and soft tissue infection, osteomyelitis, and septic arthritis. It has emerged as a significant public health threat worldwide. There is an urgent necessity to develop novel antimicrobial agents to combat MRSA infections [38]. Antimicrobial peptides have garnered attention in recent years due to their potential clinical applications and their ability to combat drug resistance [39]. In addition to direct antibacterial effects, antimicrobial peptides also have a variety of biological activities, such as inducing angiogenesis, promoting wound healing, and regulating immune response. Therefore, they have important potential value in the treatment of multi-drug-resistant bacterial infections (such as wound infection, pneumonia, sepsis, etc.) [40]. Liang, C. et al. encapsulated the tick-derived antibacterial polypeptide in methyl propylene gelatin (GelMA) hydrogel containing MXene nanoparticles and found that it could effectively promote the growth and adhesion of fibroblasts, exhibit antibacterial activity, reduce inflammation, and accelerate wound healing [41]. Xu et al. adopted a de novo design strategy to obtain specific antimicrobial peptides targeting MRSA, which can destroy MRSA cell walls and membranes, eliminate mature biofilms, and demonstrate ideal biocompatibility, systemic distribution efficacy, and immunomodulatory activity in vivo [42]. According to the Oxford Cup double-layer agar diffusion method, the antibacterial zone diameter of PFAP-1 against MRSA exceeded 15 mm (15.99 ± 0.02 mm) (Figure 5). Einipour, S.K. et al. reported that a wound dressing incorporating vancomycin nanoparticles into silk fibroin protein solution exhibited an antibacterial zone diameter of 12 mm against MRSA [43]. Similarly, Wang, F. et al. found that the antibacterial zone diameter of Australian propolis ethanol extract against MRSA was 19.7 mm [44]. These findings suggest that PFAP-1 possesses potent antibacterial activity and holds promise as a novel anti-MRSA drug. The MBC/MIC ratio of a drug reflects its antibacterial efficacy. A ratio of 1-2 indicates a bactericidal effect, while a ratio ≥4 signifies a bacteriostatic effect [45]. In this study, the MIC of PFAP-1 against MRSA was 37.5 μg/mL, the MBC was 150 μg/mL, and the MBC/MIC ratio was 4, indicating that PFAP-1 acted as a bacteriostatic agent. Compared to the control group, MRSA growth was significantly inhibited by PFAP-1 at concentrations of 75 and 150 μg/mL. At 37.5 μg/mL, PFAP-1 entered a stable phase at 4 h, maintaining a lower growth level for an extended period. These results demonstrate the dose-dependent inhibitory effect of PFAP-1 on MRSA.

As has long been known, antimicrobial peptides mainly target and destroy bacterial cell membranes or cell walls, causing the leakage of substances such as potassium ions, proteins, nucleic acids, etc., or binding with bacterial DNA to disrupt gene expression and eliminate bacteria through a highly intricate mechanism; alternatively, some antimicrobial peptides may function by activating and recruiting host immune cells or by altering toll-like receptors for microbial recognition [46,47]. Studies have shown that a β-polypeptide polymer can disrupt the cell membrane, penetrate the interior of bacteria, interact strongly with DNA, induce the production of reactive oxygen species (ROS), and further damage the bacterial membrane to eradicate bacteria. Fluorescent dye tests revealed that propidium iodide (PI) dye could penetrate the interior of bacteria, while SEM results indicated shrinkage and dimming of the bacterial cell membrane [48]. Japonicin-2LF, an antimicrobial peptide derived from the skin of *Limnonectes fujianensis*, compromised the membrane of MRSA, altered its permeability, and exhibited potent antibacterial activity [49]. The ultrastructure of cationic polypeptides R9F2 (H2N-Arg9-Phe2-C(O)NH2) and (KFF)3K (H2N-(Lys-Phe-Phe)3-Lys-C(O)NH2) on *Staphylococcus aureus* was observed using transmission electron microscopy. It was found that these two peptides can cause significant damage to the bacterial cell membrane within 1 min, leading to thinning of the cell wall and the appearance of structural disorder [50]. In this research, 4′,6-diaminidine-2 phenylindole (DAPI)/PI fluorescence staining demonstrated that the PFAP-1 recombinant protein significantly impaired the MRSA cell membrane, enabling PI dye penetration into the cell and emitting a distinct red fluorescence. Similarly, SEM results revealed that the PFAP-1 recombinant protein induced morphological changes in MRSA cells, such as cracks and pits (Figure 7 and Figure 8). However, additional evidence is necessary to ascertain whether the demise of MRSA is due to membrane destruction. It is plausible that membrane damage could be a consequence of bacterial death rather than the primary cause of death.

Biofilm formation is an organized process of microorganisms that produce extracellular polymers to resist adverse conditions. Biofilm bacteria exhibit significant drug resistance. Antimicrobial peptides have unique advantages in inhibiting biofilm formation and eliminating biofilm, without easily developing drug resistance. They provide an effective treatment for pathogenic bacterial infections [51,52]. For instance, Sun, C. et al. reported that AMP-17, a novel antimicrobial peptide extracted from *Musca domestica*, had a strong inhibitory effect on both the formation and pre-formed biofilm of *Candida albicans* [53]. Schuch, R. et al. reported that a bacteriophage, CF-301, can significantly inhibit biofilm formation of *S. aureus* on various surfaces such as polystyrene, glass, surgical mesh, and catheters. In catheters, CF-301 can eliminate all mature biofilm within 1 h and rapidly kill all released bacteria [54]. In this study, all concentrations of recombinant PFAP-1 protein could significantly inhibit the formation of MRSA biofilm. Although the dose-dependent effect was not obvious, the highest concentration of recombinant PFAP-1 had the highest absorbance, possibly due to residual PFAP-1. All concentrations of recombinant PFAP-1 protein could effectively eliminate mature MRSA biofilm in a dose-dependent manner. The results indicated that recombinant PFAP-1 could significantly inhibit MRSA biofilm, invasion, and transmission, and enhance the therapeutic effect of other therapeutic agents on MRSA. However, antimicrobial peptides from natural sources also face challenges such as weak biological activity, high potential hemolytic activity, high toxicity, and poor stability at higher concentrations, which significantly limit their practical application [55]. For example, Lung, F.D. et al. developed an effective antimicrobial peptide MAP-0403, but it exhibited high hemolytic activity, with 100% hemolytic activity against human red blood cells at 100 μM [56]. Similarly, melittin, known for its strong antibacterial and other biological activities, also demonstrates strong hemolytic activity [57]. Interestingly, although recombinant PFAP-1 in this study exhibited strong hemolytic activity at 300 μg/mL, its hemolytic activity was remarkably low (11.21%) at a bactericidal concentration of 150 μg/mL, and almost negligible at 75 μg/mL (Figure 10). This indicates its high potential for treating MRSA infections.

In addition to antibacterial activity, many bioactive peptides may also have other biological activities, such as immune regulation, anti-biofilm, antioxidant, anti-tumor, antiviral, anti-inflammatory, and angiotensin-converting enzyme activity. For example, Akbarbaglu, Z. et al. found that apricot kernel low molecular weight polypeptides have ACE inhibition, antioxidant, and antibacterial activities [58]. Zhou, J. et al. found that the head proteolytic products of *Penaeus japonicus* had significant antibacterial and ACE inhibitory activities [18]. Similarly, in addition to the significant antibacterial and anti-biofilm activities of recombinant PFAP-1 in this study, we also found that it has strong antioxidant activity. It can significantly scavenge DPPH free radicals, with an IC_50_ of 40.83 μg/mL (3.77 μM). In comparison with vitamin C in this study (with IC_50_ values of 1.11 μg/mL or 6.28 μM), its ability to scavenge DPPH free radicals appears to be slightly better. Noman, A. et al. utilized protease to hydrolyze Chinese sturgeon protein, resulting in two low-molecular-weight antioxidant peptides. These peptides exhibited the highest anti-free radical activity, with DPPH IC_50_ values of 2.59 and 2.31 mg/mL, respectively [59]. Villasenor, V.M. et al. reported that the protein hydrolysate of Mexican grasshopper powder had anti-inflammatory and antioxidant potential, with a DPPH IC_50_ of 0.78 mg/mL [60]. It can be observed that the PFAP-1 recombinant protein is similar to other reported peptides and also exhibits specific antioxidant activities. In this study, it also significantly inhibited ACE2 activity with an IC_50_ of 5.66 μg/mL or 0.52 μM. Two positive controls, MLN-4760 and DX600, were provided in the ACE2 inhibition kit we used, both of which are effective and selective human ACE2 inhibitors. The IC_50_ values in this kit were 7.5 nM and 0.15 μM, respectively, indicating that the IC_50_ of PFAP-1 recombinant protein was about 70 times and 3.5 times higher than that of the positive controls, respectively. Compared with the positive control group, it seems that its ACE2 inhibitory activity was not superior. The ACE inhibitory peptide ARL/I was discovered by Zhou, J. et al., with an IC_50_ of 125.58 μM [18]. Li et al. isolated and purified two peptides with significant ACE inhibitory activity (SNHANQLDFHP and PVQVLASAYR) from pumpkin seed meal. Their IC_50_ values were 172.07 and 90.69 μM, respectively [61]. Therefore, compared with the active peptide, PFAP-1 recombinant protein also has significant ACE2 inhibitory activity and can be used as a lead active substance for ACE2 inhibition, which can be further optimized to improve its activity. At the same time, PFAP-1 and ACE2 have a strong binding ability through molecular docking, which may change their spatial conformation and inhibit the activity of the ACE2 enzyme.

In general, although the recombinant PFAP-1 protein exhibits certain antibacterial, antioxidant, and ACE2 inhibitory activities, it is necessary to comprehensively evaluate its amino acid sequence and further investigate its structure–activity relationship. This will enable the prediction and selection of derived peptides with enhanced biological activity, reduced hemolytic activity, lower toxicity, and increased stability. Additionally, it is important to study its biological activity post-chemical synthesis, or in combination with traditional antibiotics, to assess potential synergistic antibacterial effects. Furthermore, investigating the development of functional materials loaded with PFAP-1 recombinant protein or its derived peptides by integrating nanomaterials or hydrogels can aid in understanding its biological activity. This understanding can be validated through animal experiments, offering a more scientific foundation for the future advancement and application of the active protein PFAP-1.

## 4. Materials and Methods

### 4.1. Materials 

The pET-30a vector and *Escherichia coli* BL21 (DE3) were purchased from Bio-Tech Bioengineering (Shanghai) Co., Ltd. (Shanghai, China); IPTG was purchased from Beyotime Biotechnology Co., Ltd. (Nantong, China); and Methicillin-resistant *Staphylococcus aureus* (MRSA, ATCC43300) was purchased from Ningbo Ming Zhou Biotechnology Co., Ltd. (Ningbo, China). All other chemicals and reagents were of analytical grade.

### 4.2. RT-PCR

The samples of *P. fucata* were collected at the Guangxi Beihai Haitaifeng Ecological Shellfish Seedling Farm. After cleaning with sterile PBS, the sample was stored in RNALater™ reagent (R0118, Beyotime, Nantong, China) for transport and then stored at 4 °C. An appropriate amount of pearl shellfish meat was cut into a sterile RNA-free mortar on a super clean bench, liquid nitrogen was added, and the sample was quickly ground. The total RNA was extracted using the RNAiso Plus Kit (9108Q, Takara, Beijing, China) and reverse-transcribed into cDNA with the PrimeScript™ 1st Strand cDNA Synthesis Kit (6110A, Takara, Beijing, China). Primers were designed based on the mRNA sequences of the candidate genes of *P. fucata* (Supplementary Table S2). Subsequently, RT-PCR was conducted using the cDNA as a template. The reaction mixture consisted of 25 μL 2× Pfu mix (P2021, Guangzhou Dongsheng Biotech Co., Ltd., Guangzhou, China), 2 μL upstream primer (10 μM), 2 μL downstream primer (10 μM), 1 μL cDNA template, and topped up with water to 50 μL. The RT-PCR reaction was carried out under the following conditions: 94 °C for 3 min, 94 °C for 30 s, 50 °C for 30 s, 72 °C for 1 min, 72 °C for 5 min, for 30 cycles. Following the reaction, agarose gel electrophoresis was performed to verify the amplification of specific products.

### 4.3. Plasmid Constructs

According to the UniProt protein database (Taxon ID: 50426), an uncharacterized protein (UniProt protein number: A0A194ALV1) was identified, and the complete open reading frame of its gene, with a total length of 252 bp, was obtained (GenBank number: GELH01000748.1). The codon optimization was performed in an *E. coli* expression host using GenSmart™ Codon Optimization (Version Beta 1.0) online software (https://www.genscript.com.cn/tools/gensmart%2dcodon%2doptimization?page=1) (Accessed on 15 May 2023), and the CAI and GC content were analyzed using JAVA Codon Adaptation Tool (https://www.jcat.de/Start.jsp) (Accessed on 15 May 2023) [62]. The optimized gene was named *PFAP-1*, and a DNA sequence containing *Nde* I and *Hin* dIII restriction enzyme sites was synthesized using a chemical method. Subsequently, we ligated it to the pET-30a vector. After double digestion and sequencing verification, the recombinant plasmid was transferred into *E. coli* BL21 (DE3) competent cells.

### 4.4. Bioinformatics Analysis

The antimicrobial peptides from *P. fucata* were predicted using CAMP (version R3, Biomedical Informatics Centre, Mumbai, India) (http://www.camp3.bicnirrh.res.in/) (Accessed on 16 May 2023) [16]. The physical and chemical properties of the PFAP-1 protein were predicted using ProtParam (https://web.expasy.org/protparam/) (Accessed on 16 May 2023) [63]. The transmembrane structure of PFAP-1 protein was predicted with DeepTMHMM (version 1.0.39) (https://dtu.biolib.com/DeepTMHMM) (Accessed on 16 May 2023). The secondary structure of PFAP-1 protein was predicted using SOPM (https://npsa-pbil.ibcp.fr/cgi-bin/npsa_automat.pl?page=npsa_sopma.html) (Accessed on 18 May 2023). The complete three-dimensional structure of PFAP-1 protein was predicted with I-TASSER (version 5.1) (https://zhanggroup.org/I-TASSER/) software (Accessed on 18 May 2023) [64], and visualized using PyMOL software (version 1.7.0.0) (Accessed on 4 June 2023).

### 4.5. Prokaryotic Expression, Purification, and Western Blot Assay

Refer to the previous research group article, slightly changed [8]. A single colony of *E. coli* containing pET-30a-PFAP-1 was selected, inoculated into Luria–Bertani (LB) liquid medium containing 50 μg/mL kanamycin sulfate, oscillated at 37 °C, and cultured to OD_600_ of 0.8. IPTG (ST098, Beyotime, Nantong, China) with a final concentration of 0.2 mM being added to induce expression at 15 °C and 37 °C for 16 h, and the bacteria were collected. After ultrasonic crushing (300 W, ultrasonic 5 s, intermittent 5 s, ultrasonic 30 min) (BILN-650Y, Shanghai Bilang Instrument Manufacturing Co., Ltd., Shanghai, China), the supernatant and precipitation were collected, and the precipitation was treated with 50 mM Tris (pH 8.0), and 300 mM NaCl containing 1% Triton X-100, 2 mM EDTA, and 5 mM DTT. The inclusion body was dissolved with 50 mM Tris (pH 8.0), 300 mM NaCl, 8 M Urea, and 20 mM imidazole buffers, while the Ni-IDA column was balanced. Finally, the target protein was eluted with balanced buffers with different concentrations of imidazole. Each elution component was collected for SDS-PAGE analysis. The high-purity components were collected and dialyzed to buffer solution [1× PBS (pH 7.4), 4 mM GSH, 0.4 mM GSSG, 0.4 mM L-Arginine, 1 M Urea] at 4 °C. After dialyzing, PFAP-1 protein was finally dialyzed to storage solution 1× PBS for 6–8 h at pH 7.4. The endotoxin was removed using a PurKine™ Endotoxin Removal Kit (Polymyxin B) (KTP2140, Abbkine Scientific Co., Ltd., Atlanta, GA, USA), and finally filtered by a 0.22 μm filter; the concentration was measured by the Enhanced BCA Protein Assay Kit (P0010S, Beyotime, Nantong, China), and frozen to −80 °C. HA Tag Mouse Monoclonal Antibody (AF2858, Beyotime, Nantong, China) was used as the primary antibody, and horseradish peroxidase-conjugated goat anti-mouse IgG (H + L) (A0216, Beyotime, Nantong, China) was used as the secondary antibody. The purified recombinant protein PFAP-1 was verified by Western blot.

### 4.6. Mass Spectrometry and Molecular Weight Assay

The molecular weight of the PFAP-1 protein was determined using the MALDI-TOF-TOF (Bruker Daltonics, Leipzig, Germany). PFAP-1 was digested with trypsin to generate peptide segments, and LC/MS was used for mass spectrometry analysis. The results were then compared and matched with a known protein database to determine the sequence information of the detected protein.

### 4.7. Antibacterial Activity Assays

#### 4.7.1. Detection of Antibacterial Activity Using the Oxford Cup Method

The antibacterial activity of PFAP-1 against MRSA was detected using the Oxford Cup double-plate method [65]. A bacterial double-layer plate (90 mm × 15 mm Petri Dish, BKMAM Biotechnology Co., Ltd., Changsha, China) containing 10^8^ CFU MRSA was prepared. The Oxford Cup (inner diameter (6 ± 0.1) mm, outer diameter (7.8 ± 0.1) mm, height (10 ± 0.1) mm, Jiangsu Sanaisi Scientific Instrument Co., Ltd., Yancheng, China) was removed after the medium solidified, and 100 µL of PFAP-1 protein was added to each well. The negative control consisted of sterile PBS of the same volume. The plate was pre-diffused at 4 °C for 3 h and then cultured at 37 °C for 18 h. The diameter of the inhibition zone was observed and measured. An inhibition zone diameter less than or equal to 7.8 mm was considered to indicate no antibacterial effect; an inhibition zone diameter greater than 7.8 mm was considered to indicate an antibacterial effect; greater than 7.8 mm and less than 10 mm was considered to indicate low sensitivity; greater than 10 mm and less than 15 mm was considered to indicate moderate sensitivity; greater than 15 mm was considered to indicate high sensitivity [66].

#### 4.7.2. Determination of MIC and MBC

In sterile 96-well plates (J00960, Shanghai Jing An Biotechnology Co., Ltd., Shanghai, China), sterile broth medium plus PBS was used as the blank control, bacteria-containing broth medium plus PBS was used as the negative control, and the experimental group was given bacteria-containing broth medium plus different concentrations of PFAP-1 recombinant protein. The final concentration of MRSA was 5 × 10^7^ CFU/mL, and the final concentration of PFAP-1 recombinant protein ranged from 300 to 0.59 µg/mL, with 3 replicates in each group. After incubation at 37 °C for 18 hs, 1.0 µL of 1% TTC was added to achieve a final concentration of 0.005%. Following further incubation at 37 °C for 3 h, the MIC was determined based on the color change in each group. Simultaneously, the absorbance at 485 nm was measured using a microplate reader (Synergy H1, BioTek, Winooski, VT, USA). According to the MIC test results, 20 μL of the liquid from the clarification hole was absorbed and coated on LB solid agar medium. The samples were then incubated at 37 °C for 24 h. Subsequently, the colony growth on the plate was observed, and the MBC was determined. All experiments were conducted in triplicate.

#### 4.7.3. Growth Curve Assay

In sterile 96-well plates, sterile broth medium plus PBS was used as the blank control, bacteria-containing broth medium plus PBS was used as the negative control, and the experimental group was given bacteria-containing broth medium plus different concentrations of PFAP-1 recombinant protein, resulting in final PFAP-1 concentrations of 1, 2, and 4× MIC, respectively. Three wells were set in each group, and the culture plate was placed in a microplate reader. The OD_600_ was measured every 2 h for 24 h. Finally, the growth curve was plotted.

#### 4.7.4. Fluorescent Staining Observation Assay

The 1 × 10^8^ CFU/mL of MRSA suspension was prepared, PFAP-1 protein was added, and its final concentration was 1× MIC. The same volume of PBS was set as the negative control group, and the bacteria were collected by oscillating culture at 37 °C at 100 rpm for 4 h and centrifugation at 5000 rpm for 5 min. The supernatant was removed by washing 3 times with PBS, and the precipitation was resuspended in a mixture of DAPI (10 μg/mL) (C0065, Beijing Solarbio Science & Technology Co., Ltd., Beijing, China) and PI (C0080, Beijing Solarbio Science & Technology Co., Ltd., Beijing, China) (10 μg/mL), incubated at 37 °C and stained for 30 min. After washing the bacteria 3 times with PBS, the excess dye was removed, and finally, the bacteria were suspended with an appropriate amount of PBS, and 10 μL bacterial solution was taken by fluorescence microscopy (DMi8, Leica, Wetzlar, Germany) to observe the bacterial staining and evaluate the effect of PFAP-1 on the membrane permeability of MRSA.

#### 4.7.5. Scanning Electron Microscopy (SEM) Analysis

A bacterial suspension with a concentration of 10^8^ CFU/mL was prepared, and PFAP-1 protein was added to achieve a final concentration of 1× MIC. PBS was used as the negative control, and the suspension was incubated at 37 °C and 100 rpm for 4 h. After centrifugation at 4000 rpm for 5 min, the supernatant was discarded, and the bacterial cells were washed with sterile saline three times, followed by centrifugation at 4000 rpm for 10 min. The bacterial cells were collected and fixed overnight at 4 °C with 1 mL of 2.5% glutaraldehyde. The samples were then rinsed three times with PBS for 15 min each time. For fixing, a 1% osmic acid solution prepared with PBS was used at room temperature and away from light for 1–2 h. Then rinsing was carried out three times with PBS for 15 min each time. The samples were dehydrated by adding 30%, 50%, 70%, 80%, 90%, 95%, 100%, and 100% alcohol successively for 15 min each time, and isoamyl acetate was added for two replacement times for 15 min each time. The sample was dried in a critical point dryer (K850, Quorum Technologies Ltd., Laughton, UK). Then, the sample was attached to the conductive carbon film double-sided tape and placed on the sample table of the ion sputtering instrument (MC1000, Hitachi, Tokyo, Japan) for spraying gold for about 30 s. Finally, the images were observed under a scanning electron microscope (SU8100, Hitachi, Tokyo, Japan).

#### 4.7.6. Biofilm Inhibition Assay

The biofilm experiment was performed as described by Jin, Y. et al., but with slight adjustments [67]. A bacterial suspension of 10^8^ CFU/mL was prepared using LB medium with a final concentration of 0.5% glucose (LB-G). The blank control group was set up by adding PBS to sterile LB-G medium. PBS was added into the bacterial suspension to set up the negative control group. In the experimental group, the PFAP-1 protein was added to the bacterial suspension to achieve final concentrations of PFAP-1 at 1, 2, and 4× MIC. The mixture was then incubated at 37 °C for 48 h. After incubation, the suspension was carefully discarded, and the 96-well plate was washed 3 times with PBS and fixed with anhydrous methanol for 30 min. The suspension was dried naturally, and 0.1% crystal violet was used for staining at room temperature for 20 min. After discarding the crystal violet, the 96-well plate was washed with sterile water until clear, excess water was removed, and then dried at 37 °C. The biofilm adhesion was observed by capturing images. Next, 33% glacial acetic acid was added, and the adhesive crystal violet fully dissolved after standing at 37 °C for 30 min. The absorbance at 595 nm was measured using a microplate reader, and the amount of biofilm was evaluated. In the biofilm elimination experiment, MRSA was pre-formed into biofilm for 48 h, and then PFAP-1 was treated for 24 h to observe its elimination effect on biofilm. The operation method was the same as above.

### 4.8. Hemolytic Activity Assay

The hemolysis of rabbit red blood cells was conducted using various concentrations of PFAP-1 protein. PFAP-1 was diluted to concentrations ranging from 300 to 0.59 μg/mL using the double dilution method with PBS, and then added to 96-well plates. PBS and 1.0% Triton X-100 were utilized as the negative and positive controls, respectively. An equal volume of 2% rabbit red blood cell suspension was added to each well, with 3 replicates in each group. The plates were then incubated at 37 °C for 1 h, followed by centrifugation at 1500 rpm for 10 min. The supernatant was transferred to a new 96-well plate, and the absorbance was measured at 450 nm to calculate the hemolysis rate. The hemolysis rate was determined using the formula: Hemolysis rate = (experimental group OD_450_ − negative control group OD_450_)/(positive control group OD_450_ − negative control group OD_450_) × 100%.

### 4.9. DPPH Free Radical Scavenging Assay

The antioxidant activity of the recombinant bioactive peptide PFAP-1 was assessed using the DPPH free radical scavenging kit (A153-1-1, Nanjing Jianjieng Institute of Biological Engineering, Nanjing, China). The blank tube contained 400 μL of 80% methanol and 600 μL of the working liquid from the kit. The test tube consisted of 400 μL of PFAP-1 and 600 μL of the working fluid, resulting in final PFAP-1 concentrations of 180, 90, 45, 22.5, 11.25, 5.625, and 0 μg/mL. Three replicate tubes were prepared. After mixing, the tubes were left at room temperature for 30 min away from light, then centrifuged at 4000 rpm for 5 min. Subsequently, 800 μL was transferred to a colorimetric dish, the zero was adjusted using anhydrous ethanol, and the absorbance at OD_517_ of each tube was measured. The DPPH free radical clearance (%) was calculated using the formula: (1 − (A_assay_ − A_control_) ÷ A_blank_) × 100%.

### 4.10. ACE2 Inhibition Assay

The ACE2 inhibitor screening kit (P0320S, Beyotime, Nantong, China) was utilized to assess the inhibitory effect of recombinant bioactive protein PFAP-1 on ACE2. The experimental setup included a blank control, 100% enzyme activity control, experimental group, and positive control. The PFAP-1 concentrations in the assay group were 16, 8, 4, 2, 1, 0.5, and 0.25 μg/mL. Following the provided instructions, all reagents were added, and the black transparent 96-well plate was incubated at 37 °C for 30 min in the dark. The excitation wavelength was set at 325 nm, and the emission wavelength at 393 nm. The ACE2 inhibition rate (%) was calculated using the formula: ACE2 inhibition rate (%) = (RFU_100% enzyme activity control_ − RFU _sample_)/(RFU_100% enzyme activity control_ − RFU _blank control_) × 100%.

### 4.11. Molecular Docking

The three-dimensional structures of ACE2 and PFAP-1 were predicted using I-TASSER. The HDCOK program (version 1.1) (http://hdock.phys.hust.edu.cn/) (accessed on 10 May 2024) was employed for molecular docking, and the structure with the best docking score was chosen as the standard result for subsequent interaction analysis. The interconnection score is calculated based on the ITScorePP or ITScorePR iterative scoring function. A more negative docking score indicates a potentially larger binding and stronger interaction of the binding model. Typically, the docking score of protein–protein complexes in the PDB is around −200. Therefore, we defined a confidence score dependent on the docking score to represent the likelihood of binding between two molecules as follows: Confidence score = 1.0/[1.0 + e^0.02 × (Docking Score+150)^]. In general, when the confidence score exceeds 0.7, the binding of two molecules is highly probable; when the confidence score falls between 0.5 and 0.7, the binding is likely; and when the confidence score is below 0.5, the binding is less probable [68]. Subsequently, we utilized Pymol software to visualize the spatial structure and binding sites of the ACE2 protein-PFAP-1 complex and analyzed the interaction pattern using LigPlot software [69].

### 4.12. Statistical Analyses 

Statistical analysis was performed using SPSS Statistics software (vision 19.0, SPSS, Inc., Chicago, IL, USA) and GraphPad Prism software (vision 8, GraphPad Software Inc.; San Diego, CA, USA), and all experiments were repeated three times. Quantitative results were expressed as mean ± standard deviation (*x* ± *s*). Data differences between pairs were compared using a *t*-test, and univariate analysis of variance (ANOVA) was conducted among multiple groups. A significance level of *p* < 0.05 was considered statistically significant.

## 5. Conclusions

In summary, the meat of *P*. *fucata*, as the main by-product after pearl extraction, is rich in bioactive peptides. However, the traditional separation and purification methods are complex and costly, and the application of bioinformatics tools can accelerate the screening for antimicrobial peptides. In this study, we mainly predicted whether the unknown functional protein within 100 amino acids in the total protein data of *P*. *fucata* might be an antimicrobial peptide. Two antimicrobial peptides were initially screened, one of which was PFAP-1 studied in this research. Physical and chemical analysis showed that it had the characteristics of an α-helical cationic antimicrobial peptide. After codon optimization, we inserted the *PFAP-1* gene into the pET-30a prokaryotic expression vector and conducted recombinant expression in *E. coli*. The recombinant PFAP-1 protein prepared had significant antibacterial activity against MRSA, with MIC and MBC of 37.5 μg/mL and 150 μg/mL, respectively, showing good antibacterial effect. It may exert an antibacterial effect by destroying the MRSA cell membrane. It can significantly inhibit the formation of MRSA biofilm and eliminate its mature biofilm, with low hemolytic activity. In addition, recombinant PFAP-1 protein also showed strong antioxidant activity, had a DPPH free radical elimination IC_50_ of 40.83 μg/mL, and significantly inhibited ACE2 activity with its IC_50_ of 5.66 μg/mL; moreover, preliminary molecular docking proved that PFAP-1 and ACE2 had strong binding ability. The effect of nine groups of hydrogen bonds and three groups of salt bridges as well as a large number of hydrophobic interactions forming a stable binding complex, may lead to ACE2 conformational change, and inhibited enzyme activity. Therefore, the new findings of this study will provide new avenues for the development of potential complementary and therapeutic agents in the field of food, cosmetics, or medicine.

## Figures and Tables

**Table 1:** The key physicochemical parameters of PFAP-1.

Physicochemical Parameters	PFAP-1
Number of amnio acids	83
Molecular weight	10,009.93 Da
Isoelectric point	10.40
Total net charge	+9
Hydrophobicity	43%

## Data Availability

The data presented in this study are available on request from the corresponding author.

## Supplementary Materials

The following supporting information can be downloaded at: https://www.mdpi.com/article/10.3390/md22080345/s1, Figure S1: Screening for antibacterial peptides from *Pinctada fucata* (A. Gould, 1850) by RT-PCR; Table S1: Bioinformatics prediction of antibacterial peptides from *Pinctada fucata* (A. Gould, 1850); Table S2: The primers used in this study.
