# Supplementary material for: Prokaryotic Expression, Purification, and Biological Properties of a Novel Bioactive Protein (PFAP-1) from Pinctada fucata
Source: Mar Drugs. 2024 Jul 27;22(8):345. doi: 10.3390/md22080345 (PMC11355117; doi:10.3390/md22080345)
Supplement: Supplementary file 1 [file marinedrugs-22-00345-s001.zip › marinedrugs-3119156-supplementary.pdf]

**Supplementary Material**

**Supplementary Table Legends**

**Supplementary Table S1** Bioinformatics Prediction of Antibacterial Peptides from *Pinctada fucata* (A. Gould, 1850)

| Serial number | UniProt Accession Numbers | Protein names | Length (aa) | $CAMPR_3$ |
|---|---|---|---|---|
| 1 | A0AA89C164 | Uncharacterized protein | 54 | AMP |
| 2 | A0AA88XVN3 | Uncharacterized protein | 58 | AMP |
| 3 | A0AA88XVN3 | Uncharacterized protein | 64 | AMP |
| 4 | A0AA89BRA6 | Uncharacterized protein | 64 | AMP |
| 5 | A0AA88XZM5 | Uncharacterized protein | 68 | AMP |
| 6 | A0AA89BNT2 | Uncharacterized protein | 72 | AMP |
| 7 | A0AA88XNL8 | Uncharacterized protein | 78 | AMP |
| 8 | A0A194AL40 | Uncharacterized protein | 79 | AMP |
| 9 | A0A194ALE0 | Uncharacterized protein | 79 | AMP |
| 10 | A0A194APD2 | Uncharacterized protein | 79 | AMP |
| 11 | A0AA89CCB6 | Uncharacterized protein | 79 | AMP |
| 12 | A0A194ANK0 | Uncharacterized protein | 80 | AMP |
| 13 | L8B2Q5 | Uncharacterized protein | 81 | 81 |
| 14 | A0A194AKN3 | Uncharacterized protein | 82 | AMP |
| 15 | A0AA88XWB6 | Uncharacterized protein | 82 | AMP |
| 16 | A0A194ALV1 | Uncharacterized protein | 83 | AMP |
| 17 | A0A194ANN4 | Uncharacterized protein | 83 | AMP |
| 18 | A0AA88Y172 | Uncharacterized protein | 84 | AMP |
| 19 | A0AA88YHX8 | Uncharacterized protein | 84 | AMP |
| 20 | A0AA88YLU7 | Uncharacterized protein | 85 | AMP |
| 21 | A0AA89BKC8 | Uncharacterized protein | 85 | AMP |
| 22 | A0AA89BUQ5 | Uncharacterized protein | 85 | AMP |
| 23 | A0AA88Y524 | Uncharacterized protein | 86 | AMP |
| 24 | A0AA88YUN7 | Uncharacterized protein | 87 | AMP |

| | | | | |
|---|---|---|---|---|
| 25 | A0AA89BX46 | Uncharacterized protein | 87 | AMP |
| 26 | A0A194ANZ1 | Uncharacterized protein | 88 | AMP |
| 27 | A0AA88YRQ0 | Uncharacterized protein | 88 | AMP |
| 28 | A0AA89C4L7 | Uncharacterized protein | 88 | AMP |
| 29 | A0A194APR2 | Uncharacterized protein | 90 | AMP |
| 30 | A0AA89C1W0 | Uncharacterized protein | 90 | AMP |
| 31 | A0AA88XCQ7 | Uncharacterized protein | 91 | AMP |
| 32 | A0AA88Y7V4 | Uncharacterized protein | 91 | AMP |
| 33 | A0AA88Y8M1 | Uncharacterized protein | 91 | AMP |
| 34 | A0AA89BQ63 | Uncharacterized protein | 91 | AMP |
| 35 | A0AA89C5B8 | Uncharacterized protein | 91 | AMP |
| 36 | A0AA89CBU8 | Uncharacterized protein | 91 | AMP |
| 37 | A0AA88YFB6 | Uncharacterized protein | 92 | AMP |
| 38 | A0AA88YLG8 | Uncharacterized protein | 92 | AMP |
| 39 | A0AA88YR87 | Uncharacterized protein | 92 | AMP |
| 40 | A0AA89C8J7 | Uncharacterized protein | 92 | AMP |
| 41 | L8B367 | Uncharacterized protein | 92 | AMP |
| 42 | A0AA88YB81 | Uncharacterized protein | 93 | AMP |
| 43 | A0AA88YB81 | Uncharacterized protein | 94 | AMP |
| 44 | A0A194ANS9 | Uncharacterized protein | 95 | AMP |
| 45 | A0AA88XD04 | Uncharacterized protein | 95 | AMP |
| 46 | A0A194AJI4 | Uncharacterized protein | 96 | AMP |
| 47 | A0AA88Y9H7 | Uncharacterized protein | 96 | AMP |
| 48 | A0AA89C1S0 | Uncharacterized protein | 96 | AMP |
| 49 | A0AA88XLE1 | Uncharacterized protein | 97 | AMP |
| 50 | A0AA88XPE4 | Uncharacterized protein | 97 | AMP |
| 51 | A0AA88XRL7 | Uncharacterized protein | 97 | AMP |
| 52 | A0AA88YQM4 | Uncharacterized protein | 98 | AMP |
| 53 | A0A194AP46 | Uncharacterized protein | 99 | AMP |

| | | | | |
|---|---|---|---|---|
| 54 | A0AA88XXT5 | Uncharacterized protein | 99 | AMP |
| 55 | A0AA88YHD2 | Uncharacterized protein | 99 | AMP |
| 56 | A0AA88YV66 | Uncharacterized protein | 99 | AMP |
| 57 | A0AA89BR89 | Uncharacterized protein | 99 | AMP |
| 58 | A0AA89BYQ8 | Uncharacterized protein | 99 | AMP |
| 59 | A0AA88YHP8 | Uncharacterized protein | 100 | AMP |
| 60 | F7J4X3 | Uncharacterized protein | 108 | AMP |
| 61 | A0A194APC7 | Uncharacterized protein | 114 | AMP |
| 62 | A0A194ANK2 | Uncharacterized protein | 115 | AMP |

**Supplementary Table S2** The primers used in this study.

| Serial number | UniProt Accession Number | Sequences | Size (bp) |
|---|---|---|---|
| F1 | F7J4X3 | 5'-CGGAATTCATGTACAGGGTAATAGTTCTAC-3' | 327 |
| R1 | | 5'-CCGCTCGAGCCAATATCTTCTAGAGCACTC-3' | |
| F2 | A0A194APC7 | 5'-CGGAATTCATGGCATCAGCTGATATATC-3' | 345 |
| R2 | | 5'-CCGCTCGAGATTCCTTGTATTGCATGAAC-3' | |
| F3 | A0A194AL40 | 5'-CGGAATTCATGAAGCTGTTTATTTTCGTTTTAG-3' | 240 |
| R3 | | 5'-CCGCTCGAGATGGATGTTACAAGTATGGCATC-3' | |
| F4 | A0A194ANK2 | 5'-CGGAATTCATGGCAGCAAGTGATATATC-3' | 348 |
| R4 | | 5'-CCGCTCGAGATTCCTTGTATTGCATGAAC-3' | |
| F5 | L8B367 | 5'-CGGAATTCATGGCAGGAGCTCTGAATATC-3' | 279 |
| R5 | | 5'-CCGCTCGAGGACTTTGTTAATGATTAGCTTTC-3' | |
| F6 | A0A194ALV1 | 5'-CGGAATTCATGGAAAATATACACAGTTTTTATC-3' | 252 |
| R6 | | 5'-CCGCTCGAGCAAGTCAAAAAGAAGTTTAGATG-3' | |
| F7 | A0A194ANS9 | 5'-CGGAATTCATGAAGATTCTCCTTATTCTTGC-3' | 287 |
| R7 | | 5'-CCGCTCGAGCTGGTGAGTGCATACACAAATTC-3' | |
| F8 | A0A194AP46 | 5'-CGGAATTCATGCTAAGGATGGATCGAATTAC-3' | 300 |
| R8 | | 5'-CCGCTCGAGTGGTTTCTTACACGCACACTG-3' | |

**Supplementary Figure Legends**

**Supplementary Figure S1** Screening for antibacterial peptides from *Pinctada fucata* (A. Gould, 1850) by RT-PCR

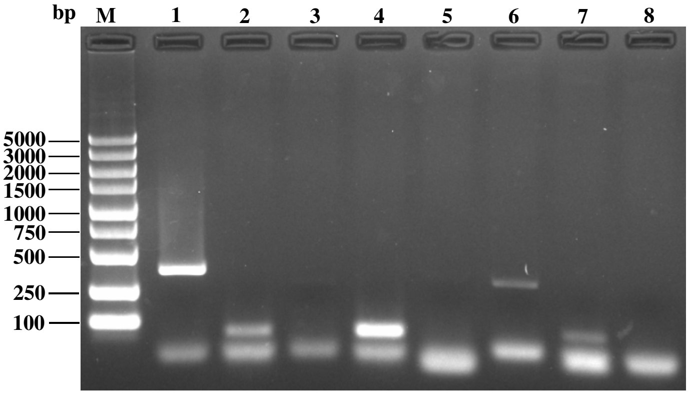


Figure S1. Screening for antibacterial peptides from *Pinctada fucata* (A. Gould, 1850) by RT-PCR. The UniProt Accession Number of No. 1 is F7J4X3. Subsequently, we named this gene *PFL-96*, and relevant work has been published [1]. The UniProt Accession Numbers 2-8 are A0A194APC7, A0A194AL40, A0A194ANK2, L8B367, A0A194ALV1, A0A194ANS9, and A0A194AP46.

**Abbreviations**

PFAP-1: a novel bioactive protein from *Pinctada fucata*; ACE2: Angiotensin-Converting Enzyme II; IPTG: isopropyl-β-d-thiogalactoside; MRSA: methicillin-resistant *Staphylococcus aureus*; MIC: minimum inhibitory concentration; MBC: minimum bactericidal concentration; $IC_{50}$: half-maximal inhibitory concentration; DPPH: 1,1-diphenyl-2-picryl-hydrazyl radical; HepG2: hepatocellular carcinomas cells; $H_2O_2$: hydrogen peroxide; *ompT*: outer membrane protease T; PBS: Phosphate Buffer Saline; ANN: Neural Network, AMPs: Antimicrobial Peptides; RT-PCR: transcription-polymerase chain reaction; CAI: the Codon Adaptation Index; SEM: Scanning electron microscopy; GelMA: methylpropylene gelatin hydrogel; ROS: oxygen species; PI: Prodium Iodide; DAPI: 4', 6-diaminidine -2 phenylindole; LB: Luria-Bertani; RBS: Ribosome Binding Site; MALDI-TOF-TOF: the matrix-assisted laser desorption ionization-tandem-time-of-flight; LB-G: LB medium with a final concentraton of 0.5% glucose; TTC: 2,3,5-Triphenyltetrazolium chloride.

## References

1. Liu, P.; Li, W.; Peng, Y.; Han, S.; Liang, Z.; Cen, Y.; Li, X.; Wang, P.; Lv, H.; Zhang, Q.; Chen, H.; Lin, J. Molecular cloning, expression, and functional analysis of a putative lectin from the pearl oyster (*Pinctada fucata*, Gould 1850). *Fish Shellfish Immunol*. **2023**, *143*, 109215.
